# Experimental warming during incubation improves cold tolerance of blue tit (*Cyanistes caeruleus*) chicks

**DOI:** 10.1242/jeb.243933

**Published:** 2022-05-25

**Authors:** Jennifer L. Page, Andreas Nord, Davide M. Dominoni, Dominic J. McCafferty

**Affiliations:** 1 Institute of Biodiversity, Animal Health and Comparative Medicine, Scottish Centre for Ecology and the Natural Environment, University of Glasgow, Rowardennan G63 0AW, UK; 2 Department of Biology, Section for Evolutionary Ecology, Lund University, SE-223 62 Lund, Sweden; 3 Institute for Biodiversity, Animal Health and Comparative Medicine, Graham Kerr Building, University of Glasgow, Glasgow G12 8QQ, UK

**Keywords:** Body temperature, Homeothermy, Thermoregulation, Endothermy, Climate change, Avian physiology

## Abstract

Climate change and increasing air temperature may alter environmental conditions for developing birds, with a range of phenotypic consequences for offspring. The thermal environment during incubation may affect the trade-off between growth and thermoregulation, but the effects of temperature on the ontogeny of endothermy are not fully understood. Therefore, we experimentally tested whether heating the nest cup of Eurasian blue tits (*Cyanistes caeruleus*) during incubation would influence cold tolerance of the chicks after hatching. Chicks from both heated and control nests showed a decrease in cooling rate with age as they became increasingly endothermic and homeothermic. However, chicks from previously heated nests cooled at a lower rate per unit surface area and from across the whole body. These chicks also had a greater body mass during the first 12 days of life compared with chicks from control nests. Lower cooling rates in heated chicks may reflect greater thermogenic capacity or a reduced surface area to volume ratio owing to a greater body mass. Future projections for climate change predict rising air temperature and increased likelihood of heatwaves, even in temperate regions. Our results indicate that nest microclimate can affect thermoregulation in offspring, and thus may be used to predict some of the future physiological responses of birds to climate change during breeding.

## Abstract (precis)

**Summary:** Chicks from experimentally heated nests demonstrate greater cold tolerance compared with control chicks when exposed to a series of post-hatch cooling challenges.

## INTRODUCTION

The warming of the Earth's climate and an increased frequency of extreme weather events (IPCC, 2021) has brought about changes to avian life cycles, from breeding phenology (Dunn and Møller, 2014; Bowers et al., 2016) to behaviour (Cunningham et al., 2015) and survival (Bourne et al., 2020). Although birds can respond to changes in weather patterns behaviourally (Wolf, 2000) and physiologically (Gerson et al., 2019; Nord and Williams, 2015), the frequent use of such responses may increase the likelihood of acute dehydration and cell damage (McKechnie and Wolf, 2010; Lin et al., 2006) or lead to missed foraging opportunities (Cunningham et al., 2013). Fitness consequences extend to young birds in the nest, which also pay a cost for thermoregulation (Andreasson et al., 2018) but may have to contend with a lower intake of food owing to reduced foraging and subsequent nest provisioning from their overheated parents (Luck, 2001; Cunningham et al., 2013; van de Ven et al., 2020). Correlative studies have demonstrated strong temperature effects on breeding birds in hot and dry climates, but less consideration has been given to birds living in temperate environments (Andreasson et al., 2020). Therefore, studies experimentally manipulating developmental conditions can be highly beneficial in aiding our understanding of how birds will respond to future environmental changes (Hepp et al., 2006; DuRant et al., 2010, 2012).

Incubation is an energetically costly stage of reproduction in birds, particularly for species that incubate without assistance from their partner (Nord and Williams, 2015). More energy is needed from the parent to keep eggs at an optimum temperature for development when air temperature is lower (Haftorn and Reinertsen, 1985), such that incubation temperature is sometimes lower in the cold (Nord et al., 2010). Thus, if energy expenditure is reduced with a warmer incubation environment, parents may have more energy available to invest in keeping eggs warm or to provision chicks more after hatching, leading to fitness benefits for chicks, such as greater immunity, body mass and condition (Reid et al., 1999; Pérez et al., 2008; Ardia et al., 2009).

Variation in egg temperature can lead to direct consequences for developing birds. For example, studies on wood ducks (*Aix sponsa*) have found a range of factors that are positively correlated with incubation temperature, such as hatching success (Hepp et al., 2006), growth rate (DuRant et al., 2010) and body and lipid mass (Hepp and Kennamer, 2012). In wild Eurasian blue tits (*Cyanistes caeruleus*; henceforth ‘blue tits’), clutches incubated at low temperature have reduced hatchability, increased developmental time and smaller chicks close to fledging (Nord and Nilsson, 2011). Furthermore, by cooling zebra finch (*Taeniopygia guttata*) eggs periodically, thus imitating variation in parental absence, embryos suffered reduced growth efficiency (Olson et al., 2006) and mass (Olson et al., 2008). Ultimately, the sum of these effects can reduce survival prospects in cold-incubated chicks (Berntsen and Bech, 2016; Hepp and Kennamer, 2012; but see Nord and Nilsson, 2016).

Mechanistically, warmer conditions during incubation may allow for accelerated maturation of the hypothalamic–pituitary–thyroid (HPT) axis, which has much influence over avian thermoregulation (Ruuskanen et al., 2021). Increased circulating levels of thyroid hormones could be associated with an upregulation of metabolic rate, which, in turn, could translate to elevated heat production capacity in response to cool temperatures after hatching. This should result in slower body cooling for chicks from warmer eggs. However, chicks that were incubated at lower temperature have also been found to have higher metabolic rates before and after hatching (Olson et al., 2006; Nord and Nilsson, 2011; DuRant et al., 2012). This has been suggested to be an epigenetic mechanism to prepare birds for future thermoregulatory demands, such that cold-incubated chicks hatch more tolerant to cold climates (Tzschentke, 2007). Other examples of pre-hatch conditioning of thermoregulation can be seen from poultry science. Studies have shown that the application of a short duration (<24 h) temperature treatment during the critical phase of embryo development (i.e. during thyroid and adrenal axis development and maturation) leads to lower corticosteroid (i.e. stress) levels when subsequently exposed to thermal challenges after hatching. This preconditioning either above (Yahav et al., 2004; Piestun et al., 2008) or below (Shinder et al., 2009, 2011) the optimal developmental temperature leads to phenotypic changes that increase chick tolerance to hot or cool temperatures post hatch. However, in the wild it is unlikely that birds would ever experience short, perfectly timed fluctuations in temperature during incubation. In fact, studies of precocial birds [those that hatch relatively independent, covered in natal down from birth (Winkler, 2016), and have some ability to thermoregulate soon after hatching (Marjoniemi and Hohtola, 1999)] suggest that experiencing slightly hypothermic conditions continuously throughout incubation, better reflecting wild incubation patterns, leads to a lower cold tolerance for chicks when compared with individuals from higher incubation temperature treatments (DuRant et al., 2012, 2013; Nord and Nilsson, 2021; reviewed by Nord and Giroud, 2020). This could perhaps be due to the slower development of muscles important for generating heat or delayed production of thyroid hormones (Visser, 1998). A reduced rate of feather development, leading to an increase in heat loss owing to a lack of insulation, offers a further explanation. We still lack knowledge of how incubation temperature influences cold tolerance in altricial chicks (those that hatch naked, with little or no capacity for endothermic thermoregulation, and are completely dependent on their parents until fledging; Winkler, 2016), particularly in free-living birds. Although in these species, parents brood their young during cold periods, chicks are sometimes unattended when the parents self-feed or forage for their offspring. If, during those periods, chicks are better at withstanding cooling or able to produce more heat, they may maximise the time spent at an optimum body temperature for growth.

We experimentally investigated whether thermal conditions of the eggs during incubation influenced the development of cold tolerance after hatching in a small songbird, the blue tit. This was done by exposing chicks from nests where the nest cup was heated, or not, during incubation to cooling challenges during the first two-thirds of the nestling period, whilst we measured changes in body surface temperature using thermal imaging. Given our wild bird model, we predicted that development will be constrained by a lower incubation temperature, as has been reported in more ecologically relevant studies (DuRant et al., 2012, 2013; Nord and Nilsson, 2021), and thus chicks from heated nests would show improved cold tolerance. Increasing nest cup temperature may indirectly influence chick phenotype by relieving parental energy expenditure; therefore, we also measured female behaviour to determine whether heated females altered their incubation pattern in response to a warmer nest microclimate. If heated females had lower energy expenditure during incubation, we predicted that this would be reflected in increased proportion of time spent incubating, with longer on-bouts and shorter off-bouts. This experimental study therefore assessed how microclimate can affect thermoregulation in offspring and provided insights into future physiological responses of birds to changes in climate in a temperate-breeding species.

## MATERIALS AND METHODS

### Study area

Fieldwork took place between March and June in 2019 and 2020 within oak (*Quercus robur*) dominated woodland at the Scottish Centre for Ecology and the Natural Environment (SCENE) in western Scotland (56.13°N, 4.61°W), where wild blue tits [*Cyanistes caeruleus* (Linnaeus 1758)] were breeding in woodcrete nest boxes (SCHWEGLER^®^, 17×26×18 cm; 32 mm entrance hole). Starting at the end of March, nest boxes were checked once a week for signs of nest building and egg laying. To determine the start of incubation (which was assumed to occur on the last day of laying), nests were visited more frequently from day 9 of egg laying (day of first egg=day 1). If nests contained fewer eggs than days of laying (for example, 8 eggs on day 9 of egg laying), it was assumed that laying had finished, and incubation had started the previous day. Air temperature (±0.1°C) was recorded every 30 min by a MiniMet Automatic Weather Station (Skye Instruments, Powys, UK) in the centre of the study area throughout the study. Mean±s.e.m. air temperature during incubation was 10.7±0.3°C in 2019 and 11.3±0.1°C in 2020. Mean environmental conditions recorded by the weather station during the experimental period were as follows: 2019: photoperiod, 05:33–21:00 h to 04:33–22:03 h; precipitation, 0.1±0.01 mm; solar radiation, 138.5±4.8 W m^−2^; wind speed, 0.4±0.01 m s^−1^; and humidity, 64.3±0.4%; 2020: photoperiod, 05:33–20:59 h to 04:33–22:04 h; precipitation, 0.1±0.01 mm; solar radiation, 171.8±5.9 W m^−2^; wind speed, 0.6±0.01 m s^−1^; and humidity, 59.8±0.4%.

### Ethics

All work involving nest disturbance was covered by licences 117614 (2019) and 156597 (2020) issued by Scottish Natural Heritage, held by D.M.D. J.L.P. was permitted to ring chicks under supervision in 2019 (licence no. T0000) and alone in 2020 (licence no. C6823) by the British Trust for Ornithology.

### Manipulation of nest cup temperature during incubation

A total of 57 nest boxes (29 heated and 28 control) were allocated to the experiment, which started on day 2 of incubation. Treatment type of the first nest was selected randomly by a coin toss and following this, treatments were allocated alternately as soon as a female began incubating. Both heated and control nest boxes had a wire mesh platform inserted underneath the nest cup, creating a space between the nest cup and the floor of the nest box. For heated treatments, two small (6×9 cm) heat packs (HotHands^®^, KOBAYASHI, Osaka, Japan) were inserted between two 1 cm thick polyethylene sheets, the purpose of which was to reduce heat loss through the nest box floor but also to prevent overheating of eggs (>40°C; Webb, 1987) owing to the heat packs. Heat packs generated heat for up to 7 h (compared with control nests) and were replaced each day (between 08:30 and 14:00 h, mean: 10:39 h) throughout the incubation period. Owing to the wire mesh platform, there was no need to move the nest cup to change heat packs. This minimised disturbance to the incubating female. Control nests were visited each day during incubation, but heat packs and polyethylene sheets were not added to the boxes to avoid altering insulation properties of the nest.

**Fig. 1.**
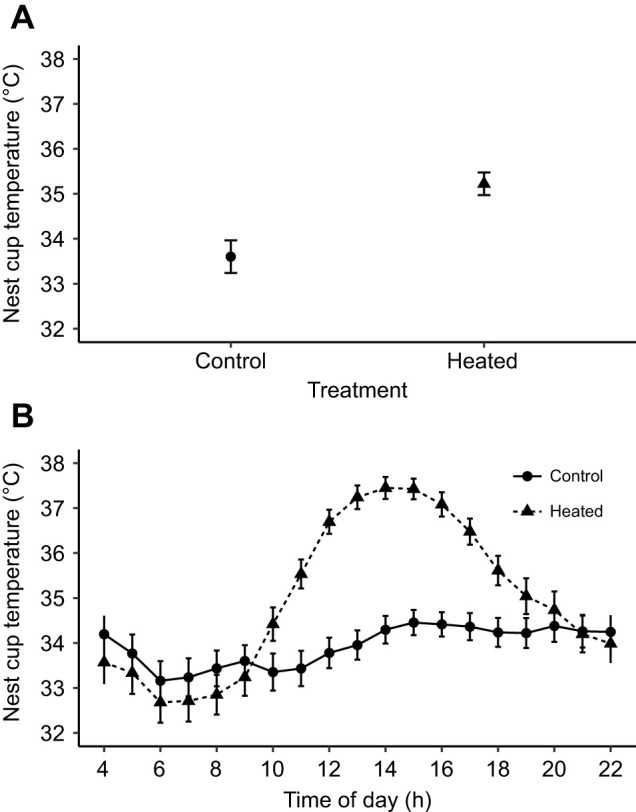
**Nest cup temperature for control and heated nests.** Data from 54 nests were used in the analysis (heated: *N*=27, control: *N*=27). Mean daily incubation temperature was calculated per nest; these values were then averaged to provide one mean temperature for each nest. (A) Global mean±s.e.m. daytime (04:00–22:00 h) nest cup temperature for control and heated nests taken from temperature loggers attached to the bottom of the nest cup. Overall, mean daytime nest cup temperature was 33.6±0.3°C for control nests and 35.2±0.3°C for heated nests. (B) Global mean±s.e.m. hourly nest cup temperature for heated nests before and after heat packs were changed daily (time of replacement 08:30 to 14:00 h, mean: 10:39 h) compared with mean hourly nest cup temperature for control nests where no heat packs were inserted.

Nest box and nest cup temperature were each recorded using temperature dataloggers (iButton^®^ DS1922-L, Sunnyvale, CA, USA; accuracy: ±0.5°C; precision: ±0.0625°C) in all nests. Nest box dataloggers were placed on the inside wall of the nest box and were programmed to record temperature every 15 min, allowing recording over the entire incubation period. Nest cup dataloggers were positioned at the bottom of the nest cup, underneath the clutch. To prevent the female removing the logger, as has been observed with dataloggers not attached to the nest (A.N. personal observation), iButton dataloggers were wrapped in thin nylon material and a section of wire was then attached to the material, passed through the bottom of the nest cup and attached to a small weight that sat on the nest box floor. Nest cup dataloggers were programmed to record temperature at 1 min intervals in 2019 and were replaced every 3 days to provide continuous measurements. In 2020, these were programmed to record at 5 min intervals. This sampling frequency allowed measurement throughout the incubation period without replacement to further minimise disturbance to the nest but was unsuitable for analysing changes to female behaviour. During daytime (04:00–22:00 h), mean±s.e.m. nest cup temperature was 1.6°C higher in heated nests (35.2±0.3°C) than control nests (33.6±0.3°C) (linear model: *P*=0.001; Fig. 1, Table 1). Over the course of 24 h, heated nest cups were 1.3°C warmer (34.9±0.3) than controls (33.6±0.3) (*P*=0.014). Daytime nest cup temperature was not affected by air temperature (*P*=0.944) or year (*P*=0.436). Nest box temperature did not differ between treatments (*P*=0.989). The purpose of the shorter sampling frequency in 2019 was to measure female incubation behaviour (see below). This allowed us to investigate whether any changes to chick phenotype caused by the heating treatment could also be explained by changes to female behaviour.

**Table 1.**
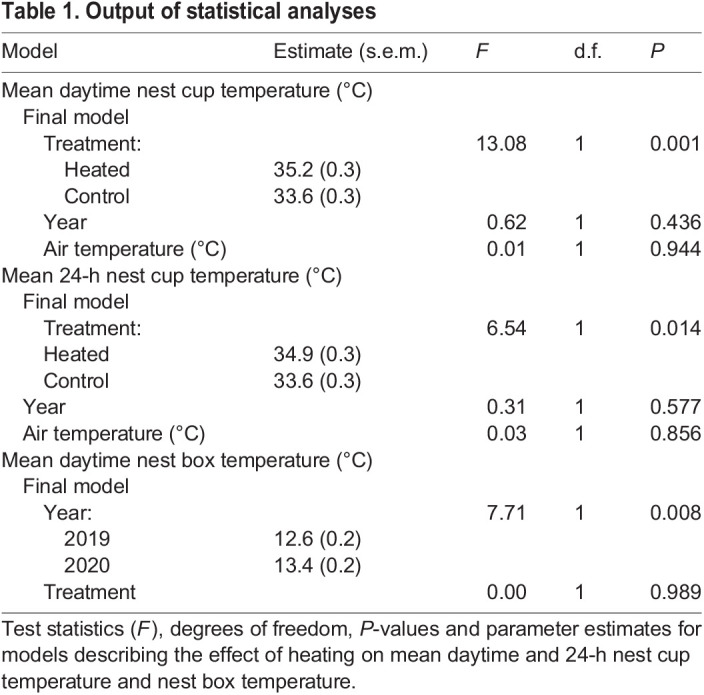
Output of statistical analyses

Hatch checks commenced 12 days after the incubation start date and continued daily until the first signs of hatching. At this point, the heating and control treatments ended, and all equipment was removed. Nests were not visited again until chicks were 4 days old, when the first cooling challenge took place.

### Incubation analyses

Data from nest cup and nest box temperature loggers in 2019 were used to quantify the number of occasions a female was away from the nest (off-bout), the number of occasions a female was present on the nest (on-bout), the duration of these occasions, and the overall percentage of time the female spent in the nest each day over the course of the incubation period, all using the ‘incR’ R package version 3.6.2 (Capilla-Lasheras, 2018). Continuous incubation was specified to take place between 22:00 and 03:00 h, the hours a bird is assumed to be incubating, to calibrate ‘incR’ functions. Parameters of ‘maxNightVariation’, ‘sensitivity’ and ‘temp.diff.threshold’ were set to optimal values of 1.5°C, 0.25 and 4°C, respectively. These values were previously found to be the most accurate when determining blue tit incubation behaviour (Capilla-Lasheras, 2018).

### Chick cooling challenge

Cooling challenges were undertaken in 5 min sessions when chicks were 4, 6, 8 and 10 days old (day of hatching=day 0). Four chicks were collected from the nest at random and placed individually inside open top plastic cups (diameter: 9 cm; rim height: 4.5 cm) covered with black matte insulating tape (emissivity: 0.98) and positioned inside a cool box. We selected four random, rather than four specific, chicks for the experiment in an effort to minimise the time a chick was out of the nest cup before the cooling challenge, because this can influence the estimated cooling rate (see Andreasson et al., 2016). In 2019, two ice packs (Thermos^®^, 16×9×3 cm) maintained temperature inside a Styrofoam cool box (30×22×20 cm) and in 2020 an electrical cool box (42×41×25 cm) (VonShef, Manchester, UK) was used. Cool box temperature (±0.1°C) was monitored throughout the challenge using a Tinytag© TK-4023 temperature logger (Gemini Data Loggers, UK), calibrated to a mercury thermometer (mean accuracy: 0.07±0.01°C). The logger recorded temperature at the start (0 min), midway (2.5 min) and end (5 min) of each challenge. Mean cool box temperature (*T*_a_) was 11.9±0.2°C in 2019 and 12.3±0.1°C in 2020 and did not differ between treatments in either year (*P*=0.901). We measured the surface temperature of the combined head and body of each chick before (*T*_1_) and after (*T*_2_) the cooling session using a thermal imaging camera (ThermaCAM E300, FLIR, ±2°C). Chicks were subsequently weighed (±0.1 g) using a digital scale and returned to their nest. The complete procedure lasted on average 9 min ±2 s (range: 6 to 13 min). We visited all nests again on day 12 to weigh all the chicks and measure tarsus (±0.1 mm) and wing (±1 mm) lengths and ring them with a uniquely numbered aluminium ring issued by the British Trust for Ornithology.

**Fig. 2.**
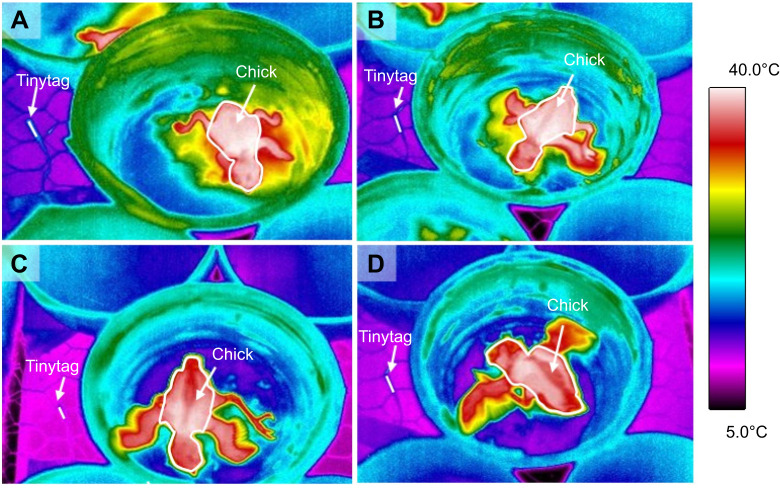
**Analysis of blue tit chick surface body temperature.** Thermal images of (A) a 4-day-old blue tit chick, (B) a 6-day-old chick, (C) an 8-day-old chick and (D) a 10-day-old chick. Using imaging software, a polygon was fitted around the body and head of each chick (wings and leg extremities have been excluded). The data inside the polygon were collected to find the average body temperature of the chick. Body temperature was calibrated by comparing the camera temperature of the Tinytag probe with the temperature taken by the probe itself at the time of the image. Camera deviations from the temperature probe were used to adjust mean body temperatures.

All thermal images were analysed using ThermaCAM Researcher Pro (Version 2.10) software (FLIR Systems), using the ‘Rain’ colour palette, with temperature scale adjusted to emphasise the outline of the chick and Tinytag probe in each image. Values for parameters known to affect the amount of radiation that reaches the camera were provided. Emissivity was set to 0.98, according to Kastberger and Stachl (2003), and distance from the camera was 50 cm. Temperature and relative humidity (%) were set according to the Tinytag inside the cool box and the automatic weather station, respectively. The region of interest (ROI) tool was used to fit a polygon around the body and head of the chick, excluding wing and legs as these were not consistently seen (Fig. 2). The mean temperature recorded from this region was calibrated using the temperature recorded by the Tinytag probe visible in each thermal image. The mean temperature of the probe taken by the thermal camera was compared with the temperature taken by the probe itself. This difference was used to correct the thermal image temperature within the ROI. The mean difference between thermal image and temperature probe was 0.5±0.01°C.

### Data analyses

Of the initial 57 nests included in the experiment, three were abandoned by parents during early incubation and thus were excluded from all datasets (heated: *N*=2, control: *N*=1). Of the 54 remaining nests, a further four were either abandoned during late incubation or died shortly after hatching and thus were excluded from cooling rate datasets (heated: *N*=1, control: *N*=3). Therefore, data from 50 nests were included in the final datasets of cold tolerance and body mass during the cooling challenges (heated: *N*=26, control: *N*=24). However, one control brood that died before day 12 measurements could be taken was excluded from the models of chick biometrics (body mass, tarsus and wing lengths) on day 12.

All statistical analyses were carried out using linear models (LMs) fitted using Base R and linear mixed models (LMMs) implemented in the ‘lme4’ package (Bates et al., 2015) in R version 3.6.2 (https://www.r-project.org/). Nest box was used as a random intercept in all LMMs to account for repeated measurements. Non-significant interactions were removed from the original model, but all main effects remained. Final models were graphically assessed for parametric assumptions using residual plots and normality histograms. If required, response variables were transformed to better meet model assumptions (see below). The ‘emmeans’ package (https://CRAN.R-project.org/package=emmeans) was used to calculate parameter estimates and s.e.m.

Surface-area-specific cooling rate of an individual chick was calculated according to Andreasson et al. (2016):
(1)

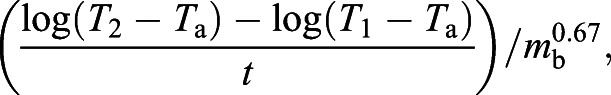
where *T*_1_ and *T*_2_ are chick temperature before and after cooling, *T*_a_ is cool box temperature, *t* is time of cooling in minutes and *m*_b_ is body mass in grams. Smaller chicks cool passively at a faster rate than larger chicks because of a higher surface area to volume ratio, which is accounted for by dividing the cooling rate by *m*_b_^0.67^. The absolute value of cooling rate was square root transformed prior to statistical analysis to meet model assumptions.

We also explored differences in whole-animal cooling using the following equation not accounting for mass:
(2)

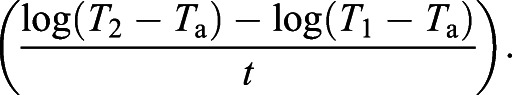


To avoid excessive cooling of chicks before the experiment started, we used averaged cooling rates across the four chicks of each cooling challenge (Andreasson et al., 2016). These values were used as the response variables in two separate LMMs with treatment, chick age, year and brood size included as explanatory variables. An interaction between treatment and chick age was also included in the original model.

Mean chick body mass was measured on post-hatch days 4, 6, 8, 10 (based on the four chicks used in the cooling challenge experiment) and 12 (based on measurement on all chicks in the brood) and was used as the response variable in a LMM with treatment, chick age, year and brood size included as explanatory variables. An interaction of treatment×age was included. Mean tarsus and wing lengths on day 12 were analysed in separate LMs with treatment and year included as fixed factors and brood size as a continuous variable.

## RESULTS

### Chick cooling rate

**Fig. 3.**
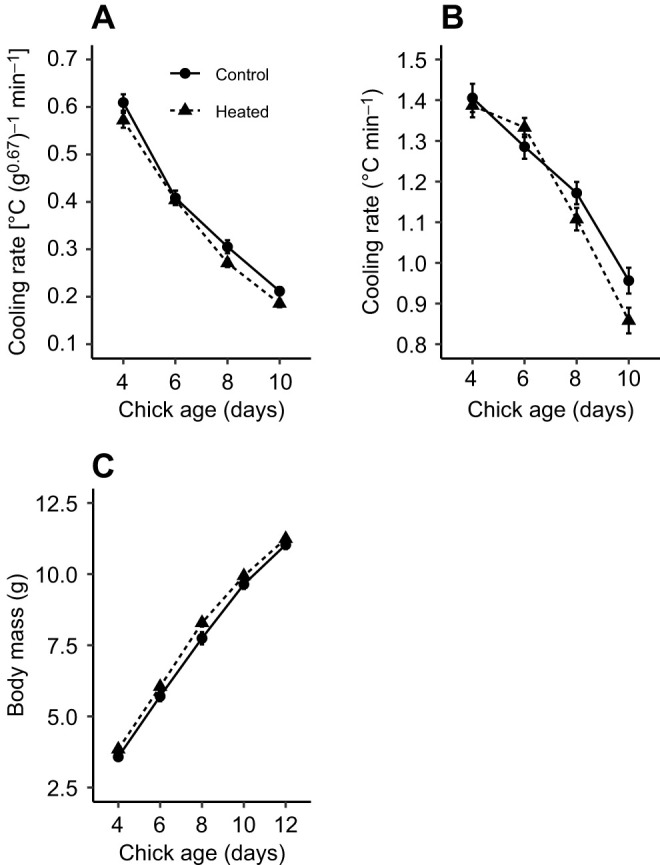
**Cooling rate and body mass of blue tit chicks from control and heated treatments**. Mean±s.e.m. (A) surface-area-specific cooling rate and (B) whole-animal cooling rate for chicks from control nests and nests that were experimentally heated during incubation on days 4, 6, 8 and 10 of age. (C) Mean±s.e.m. body mass for control and heated chicks on days 4, 6, 8, 10 and 12 of age. For cooling rate, logarithms of the data presented in A and B were used in analysis and absolute values were square root transformed prior to analysis (Eqns 1 and 2).

Parameter estimates and test statistics are presented in Table 2. The reduction in surface-area specific cooling rate with age did not differ between treatments (treatment×age interaction: *P*=0.380). There was a significant difference in the rate of cooling between treatments, with chicks from control nests losing heat 8% faster than chicks from heated nests over the course of the cooling challenge (*P*=0.036; Fig. 3A). Chick age strongly influenced the rate of temperature change, with less cooling from day 4 to day 10 of age (*P*<0.001; Fig. 3A). Chick cooling rate was 9% faster in 2020 than in 2019 (*P*=0.003). Brood size did not influence the surface-area-specific cooling rate in chicks (*P*=0.496).

**Table 2.**
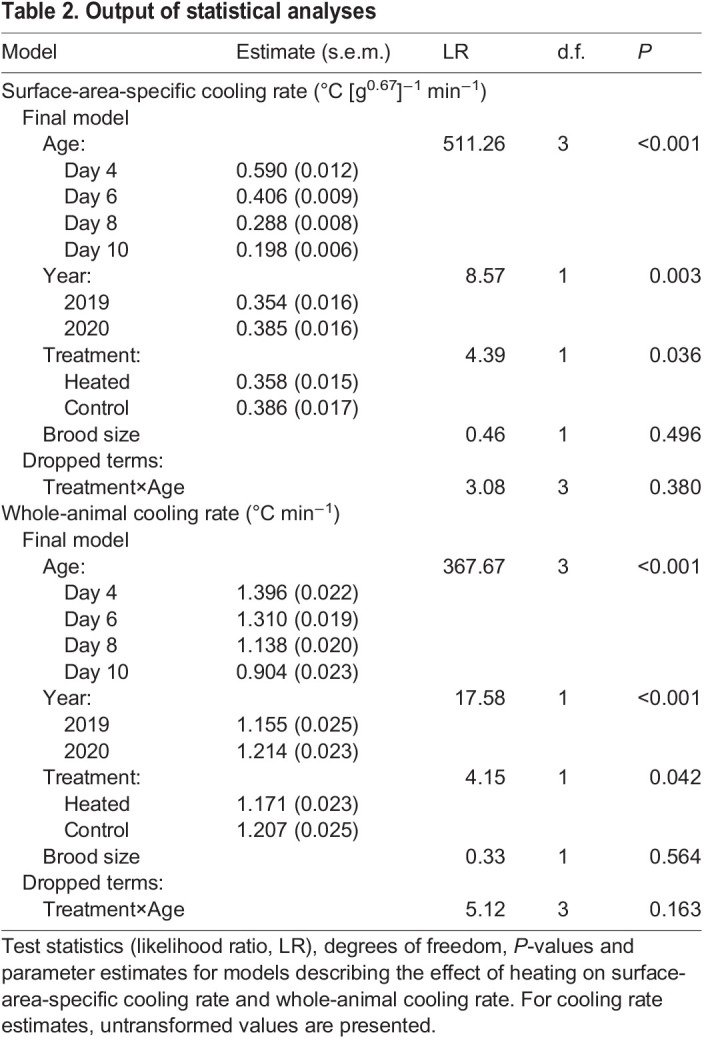
Output of statistical analyses

The reduction in whole-animal cooling rate with age did not differ between treatments (treatment×age interaction: *P*=0.163). However, chicks from control nests lost heat 3% faster than chicks from heated nests over the course of the cooling challenge (*P*=0.042; Fig. 3B). Chicks became more resistant to cooling with age (*P*<0.001; Fig. 3B) and cooling was 5% faster in 2020 than in 2019 (*P*<0.001). Brood size did not influence whole animal cooling rate in chicks (*P*=0.564).

### Chick biometrics

Parameter estimates and test statistics are presented in Table 3. The increase in body mass with age did not differ between heated and control nests (treatment×age interaction: *P*=0.251). However, chicks from heated treatments were heavier (7.9±0.1 g) than chicks from controls (7.5±0.1 g) when comparing body mass across all ages (*P*=0.033; Fig. 3C). Chick mass increased with age (*P*<0.001; Fig. 3C) and was positively influenced by brood size (*P*<0.001) but was not affected by year (*P*=0.186). The treatment did not affect wing or tarsus length (both *P*>0.05).

**Table 3.**
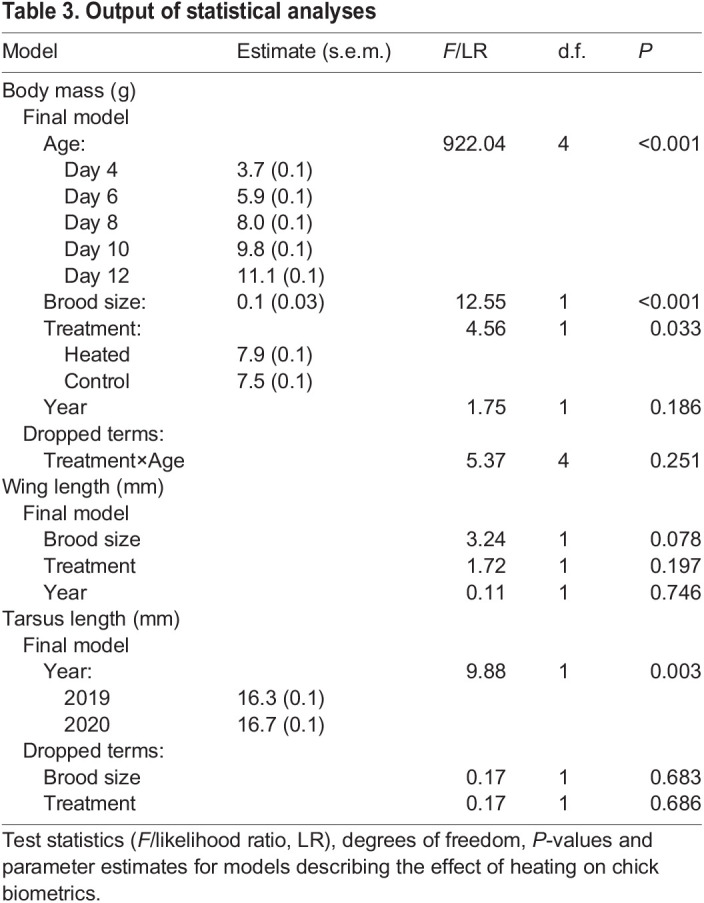
Output of statistical analyses

### Incubation behaviour

All parameter estimates and test statistics for the incubation behaviour models are presented in Table S1. Both on-bout and off-bout duration were longer for heated females (47.4±1.8 and 6.6±0.3 min, respectively) than for control females (42.5±1.6 and 5.7±0.3 min, respectively) (on-bout: *P*=0.038; off-bout: *P*=0.033). There was no effect of the experiment on the number of on-bouts (*P*=0.101) or off-bouts (*P*=0.101) in the day, or on the percentage of time the female spent on the nest (*P*=0.867).

## DISCUSSION

We provide experimental evidence that higher nest cup temperature during incubation led to a slower cooling rate when chicks were faced with a cold challenge after hatching. Moreover, chicks from heated nests were heavier than controls, suggesting that warmer temperature during incubation can also improve chick condition. The experimental treatment increased mean daytime nest cup temperature by 1.6°C. Similar temperature differences between treatments are known to affect various aspects of offspring metabolic phenotype (Nord and Nilsson, 2011; DuRant et al., 2012; Hope et al., 2018), including cold tolerance (DuRant et al., 2012, 2013; Nord and Nilsson, 2021). It is interesting to note that the temperature manipulation in this study is close to the extremes of long-term temperature increases in Scotland if greenhouse gas emissions continue to rise (Adaptation Scotland, 2022). Therefore, observed effects are relevant in predicting how future changes in weather may affect breeding birds in temperate climates.

The rate of cooling decreased with age in both treatments, but heated chicks had a slower cooling rate than control chicks, suggesting that higher incubation temperature can lead to an improved cold tolerance in offspring. This supports results from other cooling challenges, where birds incubated at higher temperatures were better equipped to deal with cold exposure (Durant et al., 2012, 2013; Nord and Nilsson, 2021). Differences in physiological development between treatments, such as in neuroendocrine pathways and accelerated maturation of the HPT axis, may provide one explanation for specific and whole-organism cooling rate results. Once activated by thermoreceptors, the hypothalamus produces thyrotropin releasing hormone (TRH), which stimulates the anterior pituitary to secrete thyroid stimulating hormone (TSH). This leads to increased production of thyroid hormones (T3 and T4), and these hormones are largely involved in regulating metabolism and body temperature (Debonne et al., 2008; Ruuskanen et al., 2021). Alternatively, because of their larger size, heated chicks may have had a greater capacity to produce heat owing to a larger amount of thermogenic tissue (Morton and Carey, 1971). Other than a change in heat producing capacity, difference in cooling rate could also be directly determined by size, which was larger in heated chicks. When altricial chicks first hatch, they are prone to high levels of heat loss, as their small size means they have a high surface area to volume ratio (Visser and Ricklefs, 1993a). Undeveloped insulation from lack of body feathers adds to this effect. As chicks age, their increased body mass leads to a decrease in surface area to volume ratio (Morton and Carey, 1971). This, and consequent growth of feathers, reduces the rate of heat loss. Therefore, body mass is one factor that aids in heat conservation of chicks (Visser and Ricklefs, 1993b). However, body mass differences between treatment groups were small. Additionally, body mass and surface area of chicks were accounted for in surface-area specific cooling rate calculations. Therefore, we believe that cooling rate results were not due solely to a difference in size between treatments and that earlier maturation of thermal physiology is a more probable factor. In line with this, heated birds were still better able to withstand cooling when we compared whole-animal cooling rate (i.e. by not accounting for surface area in the calculations), though to a lesser extent than when surface-area specific cooling rate was considered.

Similar to our results, Nord and Nilsson (2021) found that Japanese quail (*Coturnix japonica*) incubated at a low temperature were lighter and cooled more quickly than birds from higher incubation treatments. Furthermore, DuRant et al. (2013) found that a higher incubation temperature produced heavier ducklings with a greater ability to thermoregulate. It is interesting that our results are broadly similar despite the fact that quail and wood duck are precocial whereas blue tit are altricial. Altricial chicks huddle together in the nest and so rarely experience situations where their body temperature deviates by more than a few degrees (Andreasson et al., 2016), whereas precocial chicks feed independently and are exposed to the elements already from hatching. It is thus easy to see how, in a precocial species, an accelerated onset of homeothermy could be beneficial, e.g. in allowing for increased foraging efficiency during low environmental temperature (Jørgensen and Blix, 1988). Although the benefits of an advanced onset of endothermic heat production in an altricial bird are likely different, our study still suggests that the prenatal temperature stimuli triggering changes to thermoregulatory phenotypes act in a broadly synergistic manner in altricial and precocial birds.

Whilst differences in chick cooling rate and mass may be a result of a change in nest cup temperature that directly affected the embryos, we cannot exclude the possibility that providing females with a potentially less constraining (i.e. warmer) environment for incubation could carry over to increased female provisioning/brooding effort after hatching (Pérez et al., 2008), which could result in heavier chicks with a greater cold tolerance. However, our measure of nest box temperature (and hence the thermal environment that incubating females experienced) showed no difference between treatments. Additionally, although our analysis of incubation behaviour found small differences in on- and off-bout durations between treatments, there was no overall effect of heating on the proportion of time the female spent in the nest (see Table S1). The fact that there were no clear differences in incubation behaviour, and no difference in the amount of time spent incubating, suggests that the effect of the experiment on nest cup and/or egg temperature was more influential in determining subsequent chick phenotype. This notion should be critically tested in future with studies involving cross fostering of chicks after hatching, to separate the effects of embryonic and maternal environments.

### Conclusions

We have shown that elevated nest cup temperature during incubation can influence chick growth and maturation, with subsequent effects on cold tolerance. Proximate explanations for slower cooling rates in heated chicks are unknown but may reflect quicker maturation of heat producing systems, a larger body mass and reduced surface area to volume ratio or the accelerated growth of feathers, which provides greater insulation.

Studies testing the direction and magnitude of avian responses to changing environmental conditions are important, as extreme weather events are predicted to increase in frequency with climate change (IPCC, 2021). Owing to the context of our study, in a temperate climate, we suggest caution against generalising these results to other locations with different environmental conditions. Small increases in air temperature may remove some thermal constraints for birds in cooler environments, but the likelihood of birds experiencing more variable weather conditions including heatwaves will increase. This may be problematic if individuals have evolved to be cold tolerant, because there are indications that tolerance of low temperature comes at the expense of resistance to heat (Schou et al., 2021 preprint). Although no negative effects of heating were observed in this study, increases in nest temperature of a similar magnitude in already hot environments could have severe negative consequences for birds (Carroll et al., 2018). We should aim to further our knowledge on how developmental conditions can shape avian phenotypes across a range of environments, by carrying out similar studies across different latitudes to determine at what point increasing temperature during incubation ceases to be beneficial and instead becomes detrimental.

## Supplementary Material

Supplementary information
