# Supplementary material for: Experimental warming during incubation improves cold tolerance of blue tit (Cyanistes caeruleus) chicks
Source: J Exp Biol. 2022 May 25;225(10):jeb243933. doi: 10.1242/jeb.243933 (PMC9206450; doi:10.1242/jeb.243933)
Supplement: Supplementary information [file jexbio-225-243933-s1.pdf]

Table S1. Output table for Linear mixed models (LMMs), describing the effect of heating on female Blue Tit incubation behaviour. The table shows test statistics (Likelihood Ratio, LR), degrees of freedom, *P*-values and estimates for models describing number of on and off bouts, bout duration and percentage of time spent incubating during the incubation period.

Model	Estimate (s.e.m.)	LR	d.f.	*P*
Number of on bouts				
**Final model:**				
Treatment		2.69	1	0.101
Number of off bouts				
**Final model:**				
Treatment		2.69	1	0.101
On bout duration (minutes)				
**Final model:**				
Treatment:		4.29	1	0.038
Heated	47.4 (1.8)			
Unheated	42.5 (1.6)			
Off bout duration (minutes)				
**Final model:**				
Treatment:		4.53	1	0.033
Heated	6.6 (0.3)			
Unheated	5.7 (0.3)			
Percentage of time spent on the nest				
**Final model:**				
Treatment		0.03	1	0.867

Journal of Experimental Biology • Supplementary information
